# Computational Inference of Gene Co-Expression Networks for the identification of Lung Carcinoma Biomarkers: An Ensemble Approach

**DOI:** 10.3390/genes10120962

**Published:** 2019-11-22

**Authors:** Fernando M. Delgado-Chaves, Francisco Gómez-Vela, Miguel García-Torres, Federico Divina, José Luis Vázquez Noguera

**Affiliations:** 1 Division of Computer Science, Pablo de Olavide University, 41013 Seville, Spain; fmdelcha@alu.upo.es (F.M.D.-C.); mgarciat@upo.es (M.G.-T.); fdiv@upo.es (F.D.); 2 Computer Engineer Department, Universidad Americana de Paraguay, Asuncion 1209, Paraguay

**Keywords:** co-expression network, lung carcinoma, biomarker discovery, ensemble network, data mining, Bioinformatics

## Abstract

Gene Networks (GN), have emerged as an useful tool in recent years for the analysis of different diseases in the field of biomedicine. In particular, GNs have been widely applied for the study and analysis of different types of cancer. In this context, Lung carcinoma is among the most common cancer types and its short life expectancy is partly due to late diagnosis. For this reason, lung cancer biomarkers that can be easily measured are highly demanded in biomedical research. In this work, we present an application of gene co-expression networks in the modelling of lung cancer gene regulatory networks, which ultimately served to the discovery of new biomarkers. For this, a robust GN inference was performed from microarray data concomitantly using three different co-expression measures. Results identified a major cluster of genes involved in SRP-dependent co-translational protein target to membrane, as well as a set of 28 genes that were exclusively found in networks generated from cancer samples. Amongst potential biomarkers, genes NCKAP1L and DMD are highlighted due to their implications in a considerable portion of lung and bronchus primary carcinomas. These findings demonstrate the potential of GN reconstruction in the rational prediction of biomarkers.

## 1. Introduction

Over the last two decades, gene networks (GNs) have become an essential tool in the field of biomedicine [1]. Such GNs are usually presented as a graph comprising nodes and rods, where nodes represent genes (or gene products) and rods represent interactions among genes [1,2]. These rods may include a numeric value or *weight* which refers to the strength of these relationships. Therefore, not only are GNs able to identify genes related to biological processes, but also the relationships among these genes, thus providing a comprehensive picture of the studied processes [3]. GNs have been widely applied in various fields such as biology, biomedicine or bioinformatics [4,5] among others.

According to the different works in the literature [1,6], GN inference algorithms lie under four main categories: co-expression, boolean networks, differential equation-based and Bayesian networks. Within this classification, co-expression networks, which are based on information theory algorithms, arise as a significantly relevant approach due to their computational simplicity and extensive use in the literature [1,7]. These kind of networks infers relationships between genes if these show similar expression patterns, regarding an entropy measure like correlation indices or mutual information approaches among others. To do so, the degree of relationship between each pair of genes is measured, and then considered valid when this degree exceeds a certain threshold. Therefore, this threshold indicates the minimum similarity level for two expression patterns to be considered significant [8].

The main measures to evaluate the degree of co-expression between two genes, according to the literature, are correlation measures such as Pearson, Spearman or Kendall coefficients [9,10]. In addition, other measures for the generation of gene networks have been widely used, such as Mutual Information [11,12]. However, co-expression networks often present a major drawback, as the inference of relationships depends entirely on the chosen measures, which may present some limitations. For instance, the inability of the above mentioned measures to detect non-linear dependencies or their dependence on the input data distribution to obtain reliable results, as in the case of Spearman and Pearson coefficients respectively [13]. In order to overcome these issues, ensemble strategies may well be a solution, as these combine different measures for the evaluation of relationships between genes [14]. Therefore, the obtained networks are more reliable than those obtained by a single measure, also providing more accurate modelling and plausible biological insights.

Ordinarily, GN inference algorithms take gene expression datasets, e.g., microarrays or RNA-Seq, as input for the generation of the gene-gene interactions [6,7,15]. These datasets have been massively generated over the last decade for the study of some type of biological process or specific disease [16], allowing the identification of relationships between DNA, RNA, proteins and other gene products. Researchers may then perform computer analysis on this type of data before checking the results in the laboratory.

In particular, one of the most studied diseases is cancer, due to its high penetrance into the global population [17]. Moreover, cancer expression data have been screened in the quest for cancer biomarkers, which can be defined as substances, structures, or processes that can be quantified in a biological sample or their products and may indicate the prognosis of a disease [18]. In particular, lung carcinoma is among the most common tumor types and it is estimated that around 85% of the cases occur due to tobacco smoking [19,20]. Regrettably, most cases are not curable, partly as a consequence of late diagnosis, which require specific medical tests such as bronchoscopy. For this reason, lung cancer biomarkers are considered of a huge importance in the early diagnosis of the disease, and many approaches have sought for non-invasive methods for their measure. For example, in Peng et al. [21], a method is proposed for the identification of lung carcinoma biomarkers in exhaled air.

In this work we present a study of human lung carcinoma gene expression samples corresponding to smoker patients by means of an ensemble co-expression algorithm. Expression data were computational and comprehensively processed in order to generated a gene co-expression network. The algorithm applied to infer the GNs consists of an ensemble strategy which combines three widely used co-expression measures in order to rate gene-gene relationships. As a result a lung carcinoma network was generated and compared to another network generated from non-cancerous lung samples also corresponding to smoker patients. The cross analysis of these networks yielded meaningful insights on the biological functions affected in both situations, assisting the identification of potentially-novel lung carcinoma biomarkers.

The rest of the paper is organized as follows: In Section 1.1 we introduce some relevant gene networks based works applied to biomedical datasets. Then we describe, in Section 2, the dataset studied and the methods used to perform this work (network inference algorithm and the analysis approaches used). The main results obtained and the discussion are detailed in Section 3 and Section 4. Finally, the main conclusions achieved are presented in Section 5.

### 1.1. Related Works

Co-expression networks have been extensively used in the literature for the analysis and study of cancer disease. For example, Aggarwal et al. [22] applied a consensus gene co-expression meta-network of gastric cancer, the second most common cause of cancer-related deaths in the world. The results suggest, at single-gene level, an interaction between the PLA2G2A prognostic marker and the EphB2 receptor. Furthermore, the network analysis also enhances the understanding of gastric cancer at the levels of system topology and functional modules. In another work, Ma et al. [23] adopted weighted co-expression networks to describe the interplay among genes for cancer prognosis. In particular the authors presented six prognosis analyses on breast cancer and lymphoma. The results presented showed that their approach can identify genes that are significantly different from those using different alternatives. Genes that were identified using this approach presented sound biological bases, better prediction performance, and better reproducibility.

In Clarke et al. [24], a weighted version of gene co-expression network is used to analyze breast cancer samples from microarray-based gene expression studies. From the several gene clusters identified, some of them were found to be correlated with clinicopathological variables, survival endpoints for breast cancer as a whole and also its molecular subtypes. Also in 2013, the paper presented by Chang et al. [25], used a weigthed co-expression network in order to identify coexpression modules associated with malignancy menginiomas, one of the most common primary adult brain tumors. The authors identified, at the transcriptome level, 23 coexpression modules from the weighted gene coexpression network. In addition, they were able to identified a module with 356 genes that was highly related to tumorigenesis.

In 2014, the work presented by Yang et al. [26] a prognosis genes analysis based on gene co-expression networks for four cancer types using data from “The Cancer Genome Atlas”. The authors performed a systematic analysis of the properties of prognostic genes in the context of biological networks across multiple cancer types. The results of this work suggested that the prognostic mRNA genes tend not to be hub genes (genes with an extremely high connectivity). On the contrary, the prognostic genes are enriched in modules (a group of highly interconnected genes), especially in module genes conserved across different cancer co-expression networks.

In 2015, Liu et al. [27] also uses a weighted co-expression network to investigate how gene interactions influence lung cancer and the roles of gene networks in lung cancer regulation. It was found that the overall expression of one of the modules identified was significantly higher in the normal group than in the lung cancer group.

Recently in 2018, the work presented by Yang et al. [28] weighted gene co-expression network analysis (WGCNA) was applied to investigate intrinsic association between genomic changes and transcriptome profiling in neuroblastoma cancer (a highly complex and heterogeneous cancer in children). The results achieved identified multiple gene coexpression modules in two independent datasets and associated with functional pathways. The results also indicated that modules involved in nervous system development and cell cycle are highly associated with MYCN amplification and 1p deletion.

Finally, in Xu et al. [29] (2019), Xu et al. study Hepatocellular carcinoma, a very common subtype of liver cancer. The authors conducted a WGCNA to identify complex gene interactions that affect prognosis. The final results identified 10 genes that have never been mentioned in hepatocellular carcinoma and that are associated with malignant progression and patient prognosis.

## 2. Materials and Methods

In this section, the dataset studied and the methods used to perform the analysis are described. To begin with, the used dataset is presented in Section 2.1. Then, the pipeline followed for the analysis of the lung cancer dataset is exposed in the following subsections. First, data preprocessing is specified in Section 2.2. Then, relevant genes were identified in differential expression analyses, as explained in Section 2.3. Afterwards, the GN reconstruction approach is addressed in Section 2.4. Finally the exploration of the inferred networks is described in Section 2.5.

### 2.1. Lung Cancer Dataset

The dataset presented in this work corresponds to a previous study by Spira et al. [30] and Gustafson et al. [31] carried out in the Boston University Medical Center. In such studies, the gene expression level of epithelial cells coming from the respiratory tract of smoker patients was globally analyzed via microarray.

The dataset in particular retrieves the expression level of 22284 genes, along 192 samples from different smoker patients. Samples were collected from airway tissue during bronchoscopies and total RNA was extracted from these. Patients were divided in three categories: those diagnosed with lung cancer (97), those not diagnosed with lung cancer (90) and those suspected to be under cancer development (5). Although based on a relatively old platform (the Affymetrix U133A array), this dataset in particular was chosen for its suitability to specifically study the underlying genetic impairment in lung carcinoma in smoker patients.

The dataset may be openly-accessed at NCBI’s Gene Expression Omnibus (GEO) database [32], dataset record: GDS2771, reference series: GSE4115. The screening platform used to obtain this data was the Affymetrix Human Genome U133A Array [HG-U133A], from which probeset information was retrieved. The available dataset at GEO was already preprocessed in accordance with the original article [30]. In conformity with this paper, the Robust Multichip Average (RMA) algorithm was used to normalize the different datasets and achieving a certain level of similarity between all technical replicates. Also, some samples were removed from the analysis due to their poor quality (Spira et al. [30], Supplementary Methods Revised).

### 2.2. Data Preprocessing

The original dataset by Spira et al. [30] and Gustafson et al. [31] was imported to RStudio (development environment in R [33]) for data treatment and adaptation to the network inference process. From the original data a subset was selected for the present study, which seeks the comparison between cancer-diagnosed and not diagnosed smokers, thus leaving patients with cancer suspect aside. This decision was made considering the short number of patients with suspected cancer (only 5 patients), as the more analogous samples available, the more robust the GN inference will be.

First, an exploratory multidimensional scaling (MDS) plot or Principal Coordinates Analysis (PCoA) of the subset dataset was performed. This type of analysis helps in the examination of the similarity level between samples, as in the case of Gruvberger et al. [34]. In this case, the classical MDS method was applied, which assumes Euclidean distances. Graphic representation was performed using the *ggplot2* R package [35].

### 2.3. Differential Expression Analysis

The starting dataset was split in order to generate two different subsets, corresponding to cancerous and non cancerous samples respectively. DEG in cancerous samples vs. non cancerous ones were estimated using the *limma* R package [36]. Basing on linear models, *limma* has been widely used for DEG analysis, yielding prominent results [37,38]. Note those samples corresponding to smoker patients that had not been diagnosed with cancer were used as a control situation.

DEG were filtered using a significance level below 0.05 and a minimum absolute log2 fold change (FC) of 0.25. Note this log2 FC corresponds to ∼20% change in gene expression. Selected *p*-values adjustment method for multiple values was FDR Benjamini Hochberg, as it generally provides a laxer filtering [39], i.e., the larger number of DEG for a same *p*-value. The resulting DEG would be extracted from the starting dataset and would be the only ones to proceed for network inference. *p*-Values were estimated for each gene and corrected with Bonferroni step-down.

DEG information, such as log2 FC, would be additionally imported to the reconstructed networks for biological interpretation purposes. This relatively low threshold was selected in order to filter a reasonable amount of implicated genes to network reconstruction.

### 2.4. Network Inference

As stated before, co-expression networks have been extensively used in the field of biomedicine. However, they present some limitations that could be overcome by means of an ensemble strategy [40]. Therefore, we applied an ensemble strategy in order to offer a robust GN reconstruction. There are different ensemble strategies in the literature to combine the different results generated such as majority voting or average [41]. For this study, the average strategy was selected due to its good performance in the literature [42].

**Figure 1:**
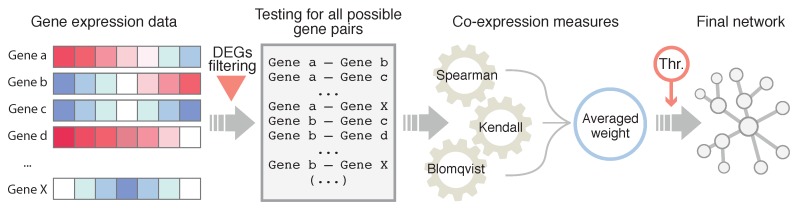
General scheme of the used inference method. For all possible gene pairs, three co-expression coefficients were calculated (Kendall, Spearman and Blomqvist) and averaged for the estimation of the final weight. Thr. refers to the thresholding step, using different co-expression indices. DEGs refer to the subset of differentially expressed genes.

A schematic representation of the GN inference approach is shown in Figure 1. For this aim, three co-expression measures were used, namely Kendall, Spearman and Blomqvist coefficients, which provide a co-expression index ranging from −1 to 1. The choice for these three measures was made after their extensive use upon GN reconstruction processes [9,13,43]. Definitions for the mentioned co-expression measures are detailed in Appendix A.

The coefficients were estimated for all possible DEG pairs both in for cancer and non cancer samples. In this way, two GNs were generated, respectively corresponding to the cancer situation and the normal situation, which can be used as a control, both under smoking conditions. Then, the average of the values obtained through each of the three coefficients is used as the final weight for the edge between each gene pair. Note that the values resulting from the application of these coefficients were also taken into consideration in the choice of these measures, as the conceived inference approach requires these values to be within a same range for latter averaging.

Finally, a threshold was established in order to keep only significant co-expressions. Thresholds varied from: 0.7, mild co-expression; to 0.8, strong co-expression; and finally 0.9, very strong co-expression. As detailed in Mukaka [44] and Cooke and Clarke [45], a cut-off of 0.5 to 0.7 (or −0.5 to −0.7) provides a moderately positive (or negative) co-expression, a cut-off of 0.70 to 0.9 (or −0.7 to −0.9) yields a high positive (or negative) co-expression and finally, a threshold of 0.9 to 1 (or −0.9 to −1), gives a very high positive (or negative) co-expression. Note that co-expressions between genes may be either positive or negative, so these thresholds are expressed as absolute values. These thresholds were defined in accordance to statistical standards [6,46,47].

Additional parameters for network representation were estimated by means of the *igraph* R package [48]. This package performs adequately with large networks and has been broadly employed in the functional analysis of biological networks [49,50]. In particular, these parameters were node degree, betweenness centrality and rank of the involved nodes. The term degree refers to the number of edges linking a particular node [51]. Those nodes comprising the largest number of relationships in a certain network are termed hubs, which according to the literature, are of a key importance in gene networks [7,52]. On the other hand betweenness centrality is defined as the addition of the fraction of all-pairs shortest paths that go through a specific node [53]. Lastly, node rank is a combination of the two previous measures. Other features such as gene IDs were also added to the nodes information table, which was imported together with the inferred networks to Cytoscape for network visualization and analysis.

### 2.5. GN Analysis: Topology and Enrichment Analyses

To perform a comprehensive analysis of the networks, we used the Cytoscape tool [54] and its apps. Cytoscape is a powerful tool to analyze GN and it is commonly used in the literature for such aim [7,55].

As the reconstructed networks were considered to be large and dense, these would be clustered using Cytoscape’s clusterMaker app [56] in order to perform an exhaustive analysis of these. The selected clustering algorithm was *GLay*, community clustering [50,57]. Clusterization enables the identification of network modules, i.e., densely-connected regions. According to the GN theory, nodes present in the same cluster are often involved in the same biological function, which will be analyzed in the following steps [58].

With the aim of exploring these functions, a Gene Ontology (GO) terms enrichment analysis was performed over the obtained clusters [59]. For this, *ClueGO* [60] & *CluePedia* [61] Cytoscape apps were used. Additional functional analyses of genes of interest were performed using *DAVID*, the Database for Annotation, Visualization and Integrated Discovery [62,63], an on-line tool for the systematic scrutiny of large lists of genes.

Finally, further infromation on the genetic disruption observed amongst potential biomarkers was revised on the GDC data portal [64] by The Genome Cancer Atlas (TGCA) [65]. The GDC portal is a data-driven platform harboring cancer data, containing information on 3,142,246 mutations registered over 22,872 genes, together with the expression level of these across 37,075 cases of different cancer types.

## 3. Results

In the following subsections, we report and discuss the main results and biological insights. Noticeably, each step of the GN reconstruction process shapes the final outcome. For this reason, the performed inference and analysis strategies are also addressed along these subsections.

### 3.1. Data Preprocessing and Exploratory Analyses

**Figure 2:**
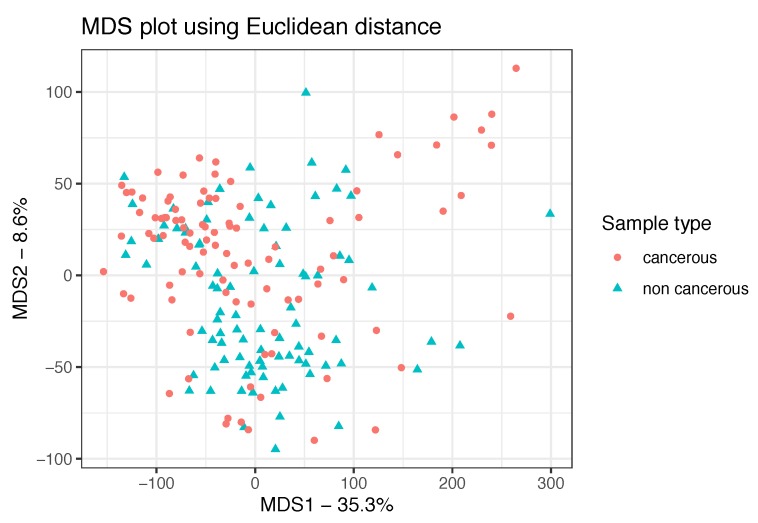
MDS/PCoA plot for the exploratory analysis of the GN inference input data. Since overlapping between sample types is significant, two groups corresponding to cancerous and non cancerous samples cannot be clearly distinguished.

MDS plots provided meaningful insights on data distribution and dataset-specific similarity level between samples. According to the performed Euclidean MDS plot, cancerous and non cancerous samples are not clearly differentiated through unsupervised analysis. MDS plot is shown in Figure 2. Although a differential gene expression pattern is suspected between cancerous and non cancerous sample types, differences were found to be fuzzy for a considerable portion of the samples, which could not be classified as part of a delimited group according to the Euclidean method used.

Notwithstanding the fact that slight dissimilarity was found between sample types, presumptive differences in gene expression profiles are thought to be responsible for the cancerous phenotype. Hence, it was assumed that all samples within a same sample type, i.e., cancerous or non cancerous, could be considered homologous. Hence, the original dataset could be split into two portions corresponding to both sample types.

### 3.2. Obtaining Differentially Expressed Genes

A total of 317 genes were identified as DEG in cancerous samples vs. non cancerous ones, in accordance with the established parameters (log2 FC > 0.25, *p*-value < 0.05). These genes were filtered from the dataset prior to GN reconstruction, so the generated networks would only comprise these. The identified DEG were considered suitable for GN inference for two reasons: (i) only the relationships between genes of interest will be modeled, and (ii) the number of genes was appropriate for latter network handling in terms of size of the final network.

**Figure 3:**
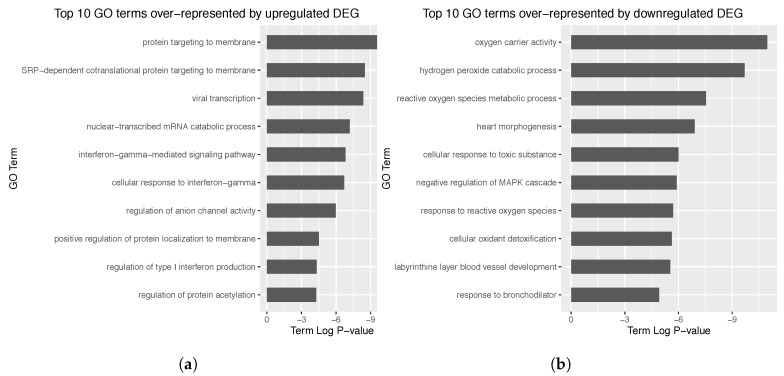
(**a**) Top 10 GO terms over-represented by the upregulated DEG. (**b**) Top 10 GO terms over-represented by the downregulated DEG. Term *p*-value was corrected with Bonferroni step-down. Note the lower the *p*-value, the more the over-represented the GO term is.

Among these DEG, 165 genes were upregulated in cancer samples when compared to control, whereas the others were found to be downregulated. Log2 FC information was added to the reconstructed networks. Strikingly, only ∼3% of DEG were differentially expressed by a 2 fold factor between sample types. Hence, gene expression levels were not found to change dramatically between cancerous and non cancerous samples. An enrichment analysis was respectively performed over the upregulated and downregulated DEG (Figure 3). As a result, upregulated DEG seemed to be involved in (possibly SRP-dependent) protein targeting to membrane (*p*-value: $1.180907\times 10^{-5}$), whereas downregulated genes appeared related to oxygen carrier activity (*p*-value: $1.744030\times 10^{-5}$). Further details on which genes are involved in the impaired biological processes upon the development of lung carcinoma will be addressed in Section 3.4.

### 3.3. GN Reconstruction and Topology Analysis

As mentioned above, two networks were inferred, corresponding to cancerous and non cancerous samples. These networks will be respectively referred as cancer and non cancer from now on for the sake of simplicity. The comparison between these networks provided meaningful biological insights on the genetic routes that were disrupted in lung carcinoma samples, as well as the impaired biological processes.

**Figure A1:**
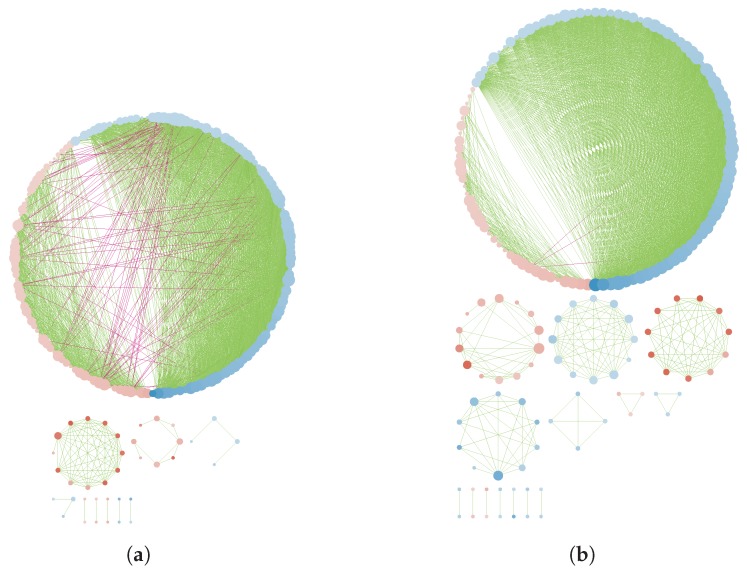
Inferred networks corresponding to (**a**) cancerous samples and (**b**) non cancerous samples, using the mild co-expression threshold (0.7). Log2 FC is represented by node color, so blue and red intensity is related to gene up or down regulation respectively. Positive co-expressions are represented in green and negative co-expressions are shown in magenta. Node size is represented according to their rank. Edge transparency is represented according to edge weight. Circle layout is represented for independent clusters. Note both networks are clustered, showing a major connected module.

**Figure A2:**
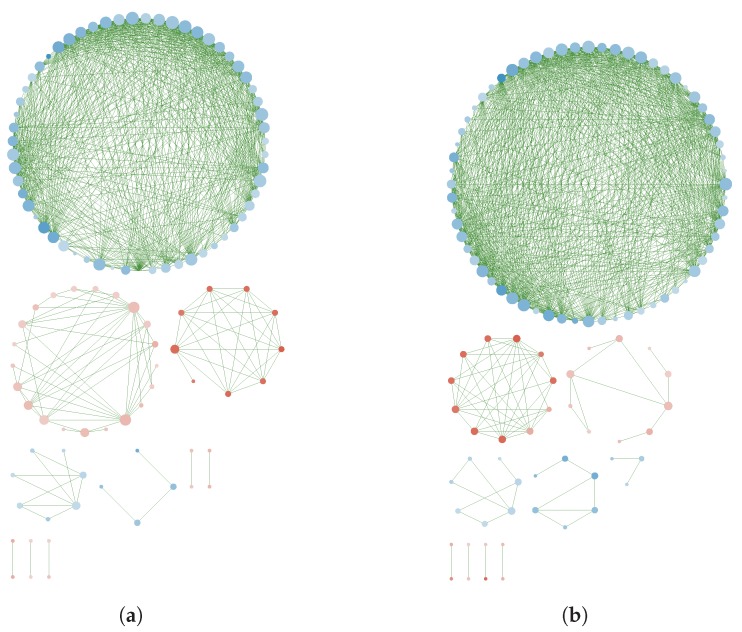
Inferred networks corresponding to (**a**) cancerous samples and (**b**) non cancerous samples, using the strong co-expression threshold (0.8). Log2 FC is represented by node color, so blue and red intensities are related to gene up or down regulation respectively. Node size is represented according to their rank. Positive co-expressions are represented in green and negative co-expressions are shown in magenta. Edge transparency is represented according to edge weight. Circle layout is represented for independent clusters. Note both networks show a major connected module.

**Figure A3:**
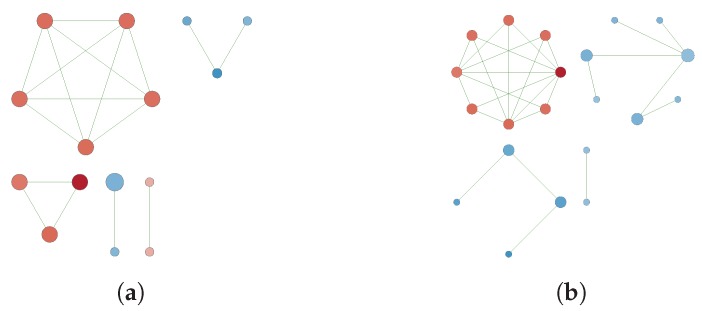
Inferred networks corresponding to (**a**) cancerous samples and (**b**) non cancerous samples, using the very strong co-expression threshold (0.9). Log2 FC is represented by node color, so blue and red intensity is related to gene up or down regulation respectively. Node size is represented according to their rank. Positive co-expressions are represented in green and negative co-expressions are shown in magenta. Edge transparency is represented according to edge weight. Circle layout is represented for independent clusters.

Among the three different thresholds that were established, the one corresponding to mild co-expression (0.7) was chosen. Other thresholds provided considerably smaller networks, which were not as informative and less suitable for latter enrichment analyses. However, the results obtained with other thresholds are addressed in the Appendix B. The cancer network comprised 197 genes and 2738 interactions, whereas the non cancer network comprised 183 genes and 2499 interactions (Appendix B, Figure A1). Networks corresponding to the strong and very strong co-expression thresholds are also shown in the Appendix B, Figure A2 and Figure A3.

Clustering analysis revealed a major cluster in both inferred networks, respectively comprising around the 70% of the nodes present in both cancer and non cancer networks. This is indicative of a main biological process being affected by DEG in cancerous vs. non cancerous samples. With this assumption, the rest of the cluster will not henceforth be considered for this study, as proposed by previous work like the one by Nepomuceno-Chamorro et al. [55].

In order to detect samples-specific genes, both networks were merged and reclustered in the so-called merged network. Although most genes are present in both cancer and non cancer networks, 28 cancer-exclusive genes were identified, as these were present in the main cluster of the cancer network, but not at its non cancer counterpart (Appendix D, Table A1). Among these, 25 showed genetic downregulation in cancer compared to non cancerous samples, whereas the three resting genes were upregulated in cancerous samples. On the other hand, 7 genes were identified as exclusively belonging to the main cluster of the non cancer network.

### 3.4. Enrichment Analysis over the Identified Network Clusters

**Figure 4:**
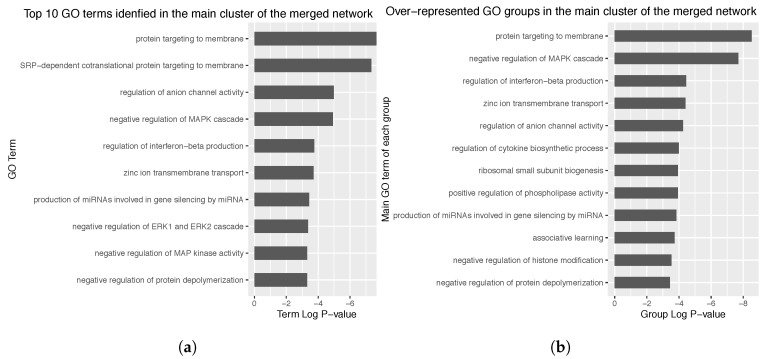
(**a**) Top 10 GO terms over-represented by the genes comprised in themain cluster of themerged network. (**b**) GO groups over-represented by the genes in the main cluster of the merged network. The main GO term of each identified group is presented as group label. Term and group *p*-value was corrected with Bonferroni step-down. Note the slower the p-value, the more the over-represented the GO term is.

**Figure A4:**
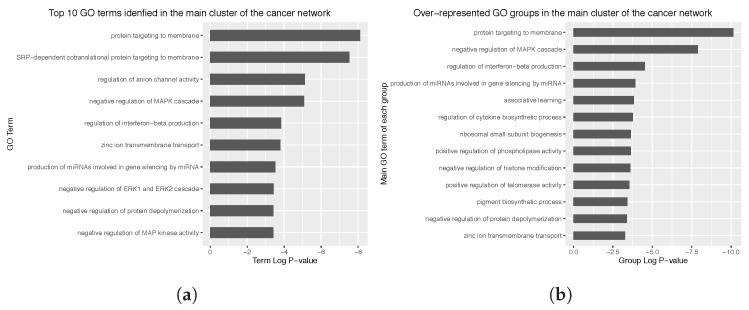
(**a**) Top 10 GO terms over-represented by the genes comprised in the main cluster of the cancer network. (**b**) GO groups over-represented by the genes comprised in the main cluster of the cancer network. The main GO term of each identified group is presented as group label. Term and group *p*-value was corrected with Bonferroni step-down. Note the slower the *p*-value, the more the over-represented the GO term is.

**Figure A5:**
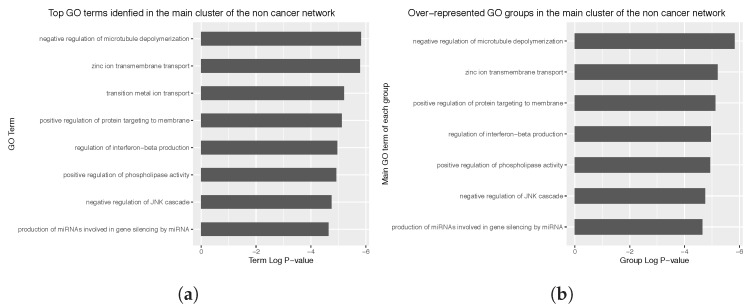
(**a**) Top 10 GO terms over-represented by the genes comprised in the main cluster of the non cancer network. (**b**) GO groups over-represented by the genes comprised in the main cluster of the non cancer network. The main GO term of each identified group is presented as group label. Term and group *p*-value was corrected with Bonferroni step-down. Note the slower the *p*-value, the more the over-represented the GO term is.

Attending to the merged network, enrichment analysis of these clusters revealed that the major cluster might be implied in protein targeting to membrane (*p*-value < 0.0005, Figure 4a). The most over-represented GO terms group is also related to this biological process (*p*-value < 0.0005, Figure 4b). Given that most genes are common between cancer and non cancer networks, and the fact that the main cluster of the merged network comprises most of these common genes, the genes involved in the reconstructed networks would be involved in the above mentioned biological functions. These analyses were also performed separately over the cancer and non cancer networks (Appendix C, Figure A4 and Figure A5).

Gene information of the 28 cancer-exclusive genes was retrieved using *DAVID* (Appendix D, Table A1). Functional analyses revealed the implication of three genes of this list in type 2 diabetes mellitus (T2DM), *p*-value: 5.6 $\times 10^{-3}$. These genes are VAMP3, HMGCR and KLF4. Interestingly enough, HMGCR is also related to lung cancer, which suggests an interplay between T2DM and lung cancer. Besides, 4/28 genes were found to be involved in enzyme regulation: HMGCR, PRPS1, PTP4A1 and SLC4A4. These processes are suggested to occur in the cytoplasm according to the functional analysis. GO enrichment analysis showed that 14/28 genes were involved in developmental processes (Appendix D, Table A2). Finally, regarding the tissue-specific genes, genes were associated with brain neoplasia (*p*-value: 4.9 $\times 10^{-4}$) and lung tissue (*p*-value: 1.0 $\times 10^{-3}$).

On the other hand, there are 7 nodes that are exclusively present at the main cluster of the non cancer network (Appendix D, Table A3). Unfortunately, some of the Affymetrix IDs could not be mapped by *DAVID*, which precluded functional analyses with this tool.

**Figure 5:**
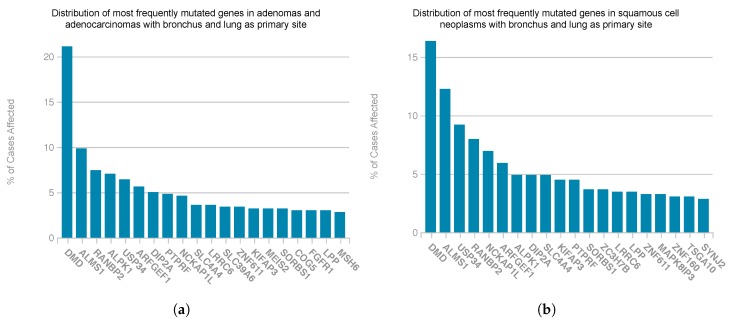
Distribution of themost frequentlymutated genes in the cases of adenomas and adenocarcinomas (**a**) and squamous cell neoplasms (**b**) registered at the GDC portal [64] presenting bronchus and lung as primary site. These genes belong to the main cluster of the reconstructed cancer network. The number of cases for adenomas and adenocarcinomas was of 497, and 489 for squamous cell neoplasms .

Finally, the observed genetic disruption was explored in the GDC portal. The 28 genes identified as cancer-exclusive were found to be affected in 7081 registered cancer cases, from which 2495 corresponded to adenomas and adenocarcinomas and 1045 corresponded to squamous cell neoplasms. Both neoplasms lie under the context of lung or bronchus carcinoma. Amongst the 28 cancer-exclusive genes, the gene NCKAP1L (NCK associated protein 1 like) was found to be affected in the 8.19% of the mentioned cases (N = 415) of lung and bronchus squamous cell neoplasms. It was also affected in the 6.15% of these cases (N = 374) of lung and bronchus adenomas and adenocarcinomas. On the other hand, when taking into consideration all genes from the main cluster of the cancer network (165), results significantly improve, as the identified gene DMD (dystrophin) is disrupted in the 21.13% of the registered cases of adenomas and adenocarcinomas with bronchus and lung as primary site, and also in the 16.35% squamous cell neoplasm cases at this same primary site, as it is shown in Figure 5. This genetic disruption was quantified in terms of simple somatic mutations (SSM), as this data was available for most cases at the GDC portal.

## 4. Discussion

Firstly, the reconstruction approach used demonstrated its efficacy in the generation of informative GNs for biomedical research. As stated in Section 2.4, these methods have been widely used for GN reconstruction and their ensemble application yielded robust inferences. The present approach was conceived as a rational biomarker discovery tool, which enables the comprehensive analysis of complex expression data to infer data that can be tested experimentally.

The utilization of DEG for GN reconstruction allowed the reconstruction of two networks, namely cancer and no cancer, which assist the modeling of the differences between sample types, thus helping in the identification of network-exclusive elements. An initial enrichment analysis was performed over DEG, in order to identify the main biological networks affected, which corresponded to the ones identified in the major clusters of the reconstructed networks.

Topology analyses revealed a major cluster for each of the two reconstructed networks. According to the literature, clustered co-expressed genes usually take part in a same biological process [15]. Taking into consideration the reconstruction approach, and the fact that DEGs were filtered prior to GN reconstruction, it can be stated that DEGs are involved in a biological process that changes between cancer and non cancer samples. The GO enrichment analysis of the cancer network’s major cluster indicated, with high significance, the involvement of these genes in SRP-dependent cotranslational protein targeting to membrane. SRP refers to signal recognition particle, which is added to nascent peptides in the endoplasmic reticulum for their latter targeting to a specific cell component. The connection between SRP and cancer histology has been previously suggested in multiple works [66,67]. For instance, in Zhong et al. [68], this GO term was found to be significantly represented by a set of DEGs which were downregulated in HER2-positive breast cancer compared to normal tissue. Also in Fahrmann et al. [69], samples non-small cell lung cancer adenocarcinoma samples were integratively analysed from metabolomic and proteomic approaches. In this work, SRP-dependent cotranslational protein to membrane was one of the top 10 most significantly disrupted pathways in cancer samples when compared to normal tissue. Taking the above into consideration, the underlying connection between SRPs and lung cancer development is yet to be clarified, but the presented approach was capable of providing a starting point for hypotheses making.

The independent reconstruction of GNs for each sample type allowed the identification of cancer and non cancer-exclusive genes. These sample type-exclusive genes could be responsible for tumor growth, potentially serving as biomarkers. Furthermore, the fact that 25/28 cancer-exclusive genes were downregulated in cancer samples compared to control normal tissue suggests the strong genetic inhibition upon cancer development. What is more, some of these cancer-exclusive genes were found to be associated with T2DM, whose implications in cancer have long been addressed [70,71,72]. It is known that cancer cells show impaired glucose metabolism, which promotes their uncontrolled proliferation and the preservation of tumor microenvironment [73]. For this reason, many newly-engineered, but also old drugs designed for other diseases such as T2DM, are used to target tumor metabolism as part of anticancer therapies [74,75]. Hence, disruptions at the genetic level can be considered either the effect or the cause of the aberrant cancer metabolism, and their deeper understanding could provide the rational design of new antitumoral drugs.

Notably, half of cancer-exclusive genes were involved in developmental processes, which could be indicative of tumor progression (Appendix D, Table A2). This GO term has also been found in previous studies, as in the case of Heller et al. [76], in which “developmental processes” was represented by tumor-specifically methylated genes in non-small cell lung cancer. Besides, 4/28 genes were found to be involved in enzyme regulation: HMGCR, PRPS1, PTP4A1 and SLC4A4. Only some of the genes in the cluster are found to be associated with the mentioned biological functions, which leads to believe that other genes within the cancer-exclusive gene list might also be involved in these processes, either directly or indirectly, but their implications might have not been discovered yet.

Furthermore, 7 genes were exclusively-found in the non cancer network (Appendix D, Table A3), which means that these genes are taking part in the processes represented in the major cluster of both networks but only in the normal situation. Besides, although these genes probably take part in the same biological process than most DEGs, the co-expressions between them were not so evident in the reconstruction process, which classified them as non cancer-exclusive genes. These genes would require further exploration as their lack in the cancer situation could also be part of cancer onset. Nevertheless, the sequences corresponding to some of these genes could not be mapped from their Affymetrix IDs using *DAVID*.

Regarding the information retrieved from the GDC portal on the potential biomarkers, the role of gene NCKAP1L in proliferation and invasion has previously been described breast and hepatocellular carcinoma [77,78]. However, poor has been described within the context of lung carcinoma, hereby suggesting potentially shared mechanisms between the three mentioned cancer types. On the other hand, the role of gene DMD, long known for its intrinsic relationship with muscular dystrophies, has previously been addressed in lung and breast cancer. In the work by Luce et al. [79], 1765 samples corresponding to 16 different non-myogenic tumors were analyzed, finding a downregulation of DMD the majority of the samples. Besides, a mutated version of DMD were observed to shorten the overall survival of patients.

Note these two identified genes were further studied because they were found to be affected in most cases of the cohort at the GDC portal. Ideally, a biomarker should be indicative and present for all cases from a same cancer type. This situation rarely occurs, being necessary to check multiple biomarkers for early cancer detection. Nevertheless, the GDC portal presents some limitations as not every gene has been tested in every sample and cancer type for SSM, so the actual affection of other identified potential biomarkers cannot be verified using this database. But even so, this leaves a door open for further experimental research, delving deeper into the implications of the suggested biomarkers, since GN are considered a powerful predictive tool.

## 5. Conclusions

In this work we presented a case of study of lung cancer by means of GN approach. To do so, the algorithm applied for inferring the GNs consists of an ensemble of three widely used co-expression measures in order to rate gene-gene relationships. As a result, two networks were generated, a lung carcinoma network and a non-cancerous lung network, both corresponding to smoker patients.

The analyses performed reveal that most DEGs between cancer and non-cancer samples were found to be associated to SRP-dependent cotranslational protein targeting to membrane. Moreover, 28 DEGs were only found in the cancer network, indicating their cancer exclusiveness. Some of these genes were associated with T2DM, developmental processes and enzyme regulation. In addition, 7 DEGs were exclusively found in the non cancer network, and their further analysis could provide further insights on their lack in the cancer situation. Finally, it is worth to mention that among DEGs present in the analyzed clusters, biomarkers exploration is possible and considered a subsequent step in this research.

Genes NCKAP1L and DMD, identified in the main cluster of the cancer network, were identified as mutated in a considerable percentage of the cases of adenomas, adenocarcinomas and squamous cell neoplasms whose primary site was bronchus and lung, and which were registered at the GDC portal by TCGA.

As future works, we will attempt to refine the process of generating the networks. To this end, we will study new measures that take into account not only linear relations of gene expression, but also non-linear relations. This is due to the fact that non linearity is a grounded assumption when it comes to gene expression [80,81]. Nevertheless, the reconstruction method provided meaningful biological insights even obviating non-linear dependencies.

## Abbreviations

The following abbreviations are used in this manuscript:

DEG	Differentially Expressed Genes
FC	Fold Change
GO	Gene Ontology
GN	Gene Networks
PCoA	Principal Coordinates Analysis
MDS	Multidimensional scaling
T2DM	Type 2 diabetes mellitus

## Appendix A. Definitions of the Used Co-Expression Measures

Co-expression measures are used in a bivariate analysis measuring the association strength between two genes, and whether this relationship is positive or negative. The presented co-expression measures take values ranking from −1 to +1. Hence, a value of +1 indicates a perfect positive association degree between two genes, whereas a value of −1, does likewise in perfect negative correlations. On the other hand, a value towards 0, is indicative of no/weak relationship.

The three chosen co-expression measures accomplish the above-mentioned features, which makes them suitable for a straight-forward ensemble strategy implementation. In the following subsections, these measures are described in mathematical detail.

### Appendix A.1. Kendall Co-Expression Measure

Kendall co-expression measure is a non-parametric hypothesis test which assess the weight of a relationship between two genes, e.g., *a* and *b*, whose expression level has been measured *n* times. Hence, the total number of pairings between *a* and *b* is n(n−1)/2.

The dataset containing all *n* expression level observations corresponding to genes *a* and *b* will look like ($a_{1}$, $b_{1}$), ($a_{2}$, $b_{2}$), ..., ($a_{n}$, $b_{n}$). Thus, for every pair of observations ($a_{i}$, $b_{i}$) and ($a_{j}$, $b_{j}$), given *j* > *i*, are considered concordant if $a_{i}$ > $a_{j}$ and $b_{j}$ > $b_{j}$, or if, $a_{i}$ < $a_{j}$ and $b_{j}$ < $b_{j}$. In the contrary case, if $a_{i}$ > $a_{j}$ and $b_{j}$ < $b_{j}$, or if, $a_{i}$ < $a_{j}$ and $b_{j}$ > $b_{j}$, the pair of observations is considered discordant [46]. Hence, Kendall co-expression measure can be estimated using the following equation:

$$\tau =\frac{Nc-Nd}{\frac{1}{2}n\left(n-1\right)}$$

Where Nc refers to the number of concordant pairs of observations and Nd to the number of discordant pairs of observations. Finally τ refers to Kendall co-expression value.

### Appendix A.2. Spearman Co-Expression Measure

Spearman co-expression measure is also a non-parametric hypothesis test which assess the degree of relationship between two genes *a* and *b*, which have been observed at their expression level *n* times. The Spearman co-expression measure does not consider any prior assumption on the data distribution and it is useful in the analysis of monotonic relationships (linear or not).

Again, datasets for each gene pair looks like *a* = ($a_{1}$, ..., $a_{n}$) & *b* = ($b_{1}$, ..., $b_{n}$). In this case, the Spearman co-expression measure acts on the ranks of the data rather than the raw data. This way, the respective ranks for both distributions, of the form ($R_{1}$, ..., $R_{n}$) and ($S_{1}$, ..., $S_{n}$), are calculated [82]. Thus, the Spearman co-expression measure can be calculated using the following formula:

$$\rho =1-\frac{\sum_{i=1}^{n}{\left(R_{i}-S_{i}\right)}^{2}}{n\left(n^{2}-1\right)}$$

Where ρ refers to Spearman co-expression value and *n* is the number of observations.

### Appendix A.3. Blomqvist Co-Expression Measure

Finally, Blomqvist co-expression measure is also a non-parametric hypothesis test for the association of two genes. This measure places the emphasis on the difference of observed values among the first ranks in the orderings induced by the variables.

Again if ($a_{1}$, $b_{1}$), ..., ($a_{n}$, $b_{n}$) represent the expression level of genes *a* and *b* across *n* measurements, a cumulative distribution function (cdf) can be defined as cdf F (a, b). Provided $\bar{a}$ and $\bar{b}$ denote the average expression level for genes *a* and *b*, let the *a*,*b* plane be divided in four areas by the lines x = $\bar{a}$ and b = $\bar{b}$. Thus, information on the co-expression of these genes can be obtained from the number of samples belonging to any of the quadrants 1 or 3 ($n_{1}$), when compared with the number of samples belonging to either the second or fourth quadrant ($n_{1}$) [83]. Blomqvist co-expression measure is then defined as:

$$B=\frac{2n_{1}}{n_{1}+n_{2}}-1-1\leq B\leq 1$$

## Appendix B. Reconstructed Networks with High Thresholding

The cancer and no cancer networks corresponding to mild co-expression (0.7) are shown in Figure A1. These networks would proceed for latter topology and enrichment analysis as preliminary analyses revealed their suitability for the goal of our study.

As mentioned in the main text, strong and very strong co-expression thresholds, respectively 0.8 and 0.9, were also used for the GN inference process. The cancer network for the strong co-expression threshold (weight cutoff: 0.8) comprised 110 nodes and 740 rods, whereas its non cancer equivalent comprised 109 nodes and 888 rods. On the other hand, the cancer network for the very strong co-expression threshold (weight cutoff: 0.9) comprised 15 nodes and 17 rods, whereas its non cancer counterpart comprised 21 nodes and 38 rods.

Notably, all co-expressions in these networks are positive. Clustering also revealed genetic interactions in the case of the 0.8 network (Figure A2). Nodes within these clusters represent around the 50% of the total number of nodes in these networks. After conducting similar analyses to the one presented with the 0.7 network, no new biological results were found for these networks compared to those already exposed.

## Appendix D. Detailed Lists of Sample Type-Exclusive Genes

Comparison of the cancer vs. the non cancer network yielded a list of 28 cancer-exclusive genes. These were submitted to the *DAVID* database for information retrieval, which is shown in Table A1. According to *DAVID* functional analysis genes are VAMP3, HMGCR and KLF4 are related to T2DM. Besides, HMGCR is also related to lung cancer.

In Table A2, the 14 cancer-exclussive genes that were found to share the GO term ’developmental process’ (GO:0032502) are listed. This GO term is related to processes resulting in the progression of subcellular structures, cells, tissues or organs from a starting situation to a final situation. This could be related to tumor progression airway epithelial cells.

**Table A1:** The 28 cancer-exclusive genes, found in the main cluster of the cancer network which were not found at its non-cancer counterpart. Regulation refers to the increase (up) or decrease (down) of the gene expression levels.

Affymetrix ID	Gene Name	Gene Description	Regulation
202539_s_at	HMGCR	3-hydroxy-3-methylglutaryl-CoA reductase	Down
211672_s_at	ARPC4-TTLL3	ARPC4-TTLL3 readthrough	Down
209288_s_at	CDC42EP3	CDC42 effector protein 3	Down
213826_s_at	H3F3A	H3 histone family member 3A	Up
220266_s_at	KLF4	Kruppel like factor 4	Down
212327_at	LIMCH1	LIM and calponin homology domains 1	Down
207480_s_at	MEIS2	Meis homeobox 2	Down
217549_at	NCKAP1L	NCK associated protein 1 like	Up
203582_s_at	SPHAR	S-phase response (cyclin related)	Down
216064_s_at	AGA	aspartylglucosaminidase	Down
201942_s_at	CPD	carboxypeptidase D	Down
203492_x_at	CEP57	centrosomal protein 57	Down
213753_x_at	EIF5A	eukaryotic translation initiation factor 5A	Up
218343_s_at	GTF3C3	general transcription factor IIIC subunit 3	Down
206483_at	LRRC6	leucine rich repeat containing 6	Down
218212_s_at	MOCS2	molybdenum cofactor synthesis 2	Down
206302_s_at	NUDT4	nudix hydrolase 4	Down
208447_s_at	PRPS1	phosphoribosyl pyrophosphate synthetase 1	Down
200730_s_at	PTP4A1	protein tyrosine phosphatase type IVA, member 1	Down
218276_s_at	SAV1	salvador family WW domain containing protein 1	Down
203908_at	SLC4A4	solute carrier family 4 member 4	Down
217975_at	TCEAL9	transcription elongation factor A like 9	Down
209149_s_at	TM9SF1	transmembrane 9 superfamily member 1	Down
204426_at	TMED2	transmembrane p24 trafficking protein 2	Down
211689_s_at	TMPRSS2	transmembrane protease, serine 2	Down
214007_s_at	TWF1	twinfilin actin binding protein 1	Down
211763_s_at	UBE2B	ubiquitin conjugating enzyme E2 B	Down
201337_s_at	VAMP3	vesicle associated membrane protein 3	Down

**Table A2:** List of 14/28 cancer-exclusive genes associated with the GO term developmental process (GO:0032502).

Affymetrix ID	Gene Name	Gene Description
202539_s_at	HMGCR	3-hydroxy-3-methylglutaryl-CoA reductase
209288_s_at	CDC42EP3	CDC42 effector protein 3
213826_s_at	H3F3A	H3 histone family member 3A
220266_s_at	KLF4	Kruppel like factor 4
207480_s_at	MEIS2	Meis homeobox 2
217549_at	NCKAP1L	NCK associated protein 1 like
203492_x_at	CEP57	centrosomal protein 57
206483_at	LRRC6	leucine rich repeat containing 6
208447_s_at	PRPS1	phosphoribosyl pyrophosphate synthetase 1
200730_s_at	PTP4A1	protein tyrosine phosphatase type IVA, member 1
218276_s_at	SAV1	salvador family WW domain containing protein 1
204426_at	TMED2	transmembrane p24 trafficking protein 2
211763_s_at	UBE2B	ubiquitin conjugating enzyme E2 B
201337_s_at	VAMP3	vesicle associated membrane protein 3

**Table A3:** The 7 non cancer-exclusive genes identified at the main cluster of the non cancer network which were not found at its cancer counterpart. Regulation refers to the increase (up) or decrease (down) of the gene expression levels. Note some Affymetrix IDs could not be mapped.

Affymetrix ID	Gene Name	Gene Description	Regulation
212206_s_at	H2AFV	H2A histone family member V	Down
209703_x_at	METTL7A	methyltransferase like 7A	Up
217734_s_at	WDR6	WD repeat domain 6	Up
215359_x_at	LOC101060181	zinc finger protein ZnFP12	Up
222339_x_at	-	-	Up
220856_x_at	-	-	Up
208082_x_at	-	-	Up
